# Chronic Stress Reduces Nectin-1 mRNA Levels and Disrupts Dendritic Spine Plasticity in the Adult Mouse Perirhinal Cortex

**DOI:** 10.3389/fncel.2018.00067

**Published:** 2018-03-13

**Authors:** Qian Gong, Yun-Ai Su, Chen Wu, Tian-Mei Si, Jan M. Deussing, Mathias V. Schmidt, Xiao-Dong Wang

**Affiliations:** ^1^Department of Neurobiology, Key Laboratory of Medical Neurobiology of Ministry of Health of China, Zhejiang Province Key Laboratory of Neurobiology, Zhejiang University School of Medicine, Hangzhou, China; ^2^National Clinical Research Center for Mental Disorders, Peking University Sixth Hospital/Institute of Mental Health, Beijing, China; ^3^Key Laboratory of Mental Health, Ministry of Health, Peking University, Beijing, China; ^4^Department of Stress Neurobiology and Neurogenetics, Max Planck Institute of Psychiatry (MPG), Munich, Germany

**Keywords:** chronic stress, corticotropin-releasing hormone receptor 1, dendritic spine, nectin-1, perirhinal cortex

## Abstract

In adulthood, chronic exposure to stressful experiences disrupts synaptic plasticity and cognitive function. Previous studies have shown that perirhinal cortex-dependent object recognition memory is impaired by chronic stress. However, the stress effects on molecular expression and structural plasticity in the perirhinal cortex remain unclear. In this study, we applied the chronic social defeat stress (CSDS) paradigm and measured the mRNA levels of nectin-1, nectin-3 and neurexin-1, three synaptic cell adhesion molecules (CAMs) implicated in the adverse stress effects, in the perirhinal cortex of wild-type (WT) and conditional forebrain corticotropin-releasing hormone receptor 1 conditional knockout (CRHR1-CKO) mice. Chronic stress reduced perirhinal nectin-1 mRNA levels in WT but not CRHR1-CKO mice. In conditional forebrain corticotropin-releasing hormone conditional overexpression (CRH-COE) mice, perirhinal nectin-1 mRNA levels were also reduced, indicating that chronic stress modulates nectin-1 expression through the CRH-CRHR1 system. Moreover, chronic stress altered dendritic spine morphology in the main apical dendrites and reduced spine density in the oblique apical dendrites of perirhinal layer V pyramidal neurons. Our data suggest that chronic stress disrupts cell adhesion and dendritic spine plasticity in perirhinal neurons, which may contribute to stress-induced impairments of perirhinal cortex-dependent memory.

## Introduction

Repeated exposure to severe stress during adulthood impairs memory and increases the risk for psychiatric disorders in susceptible individuals (de Kloet et al., 2005; Lupien et al., 2009; Chattarji et al., 2015; Duman et al., 2016). Chronic stress, acting through stress mediators including glucocorticoids and corticotropin-releasing hormone (CRH), causes structural modifications and functional abnormalities in hippocampal neurons, which collectively contribute to memory deficits (Joëls et al., 2007; Joëls and Baram, 2009; Maras and Baram, 2012).

As a major component of the parahippocampal region, the perirhinal cortex has both indirect and direct connections with the hippocampus (van Strien et al., 2009; Kealy and Commins, 2011) and plays an integral role in several key aspects of memory, especially recognition memory (Eichenbaum et al., 2007; Squire et al., 2007; Suzuki and Naya, 2014). Excitotoxic lesions of the perirhinal cortex lead to a selective loss of novel object recognition memory (Barker et al., 2007; Barker and Warburton, 2011), which is also compromised in animals subjected to chronic adult stress (Wang et al., 2011a; Barsegyan et al., 2015; Franklin et al., 2018) or early-life stress (Reincke and Hanganu-Opatz, 2017). Intriguingly, lesions of the perirhinal cortex buffer acute stress-induced anxiety-related behavior (Schulz-Klaus, 2009), further indicating its involvement in stress-induced behavioral and cognitive deficits. However, in contrast to the extensive studies on the stress effects on the hippocampus, evidence concerning the impact of chronic stress on the perirhinal cortex and the underlying mechanisms is still sparse.

Trans-synaptic cell adhesion molecules (CAMs) participate in the development, maintenance and remodeling of synapses (Dalva et al., 2007; Missler et al., 2012). CAMs are modulators of the stress effects on brain and memory (Sandi, 2004). Recently, two families of CAMs, nectins and neurexins, have been linked to stress-induced memory deficits (Wang et al., 2011a; van der Kooij et al., 2014). Chronic adult stress or early-life stress reduces hippocampal levels of nectin-3 and neurexin-1 in a CRH receptor 1 (CRHR1)-dependent manner (Wang et al., 2011a,b; Liao et al., 2014). Moreover, suppression of hippocampal nectin-3 levels reduces dendritic spine density and impairs long-term spatial memory, while overexpression of hippocampal nectin-3 attenuates stress-induced spine loss and memory deficits (Wang et al., 2013; van der Kooij et al., 2014). Nonetheless, it remains unknown whether nectins and neurexins in the perirhinal cortex are also influenced by chronic stress.

In the present study, we investigated the effects of chronic social defeat stress (CSDS) on the mRNA levels of nectin-1, nectin-3 and neurexin-1 in the mouse perirhinal cortex, and examined the involvement of the CRH-CRHR1 system in the stress effects. Moreover, we analyzed the density and morphology of dendritic spines in perirhinal layer V pyramidal neurons in control and stressed mice. We hypothesized that chronic adult stress would dysregulate the mRNA levels of CAMs in the perirhinal cortex, which might be modulated by the CRH-CRHR1 system. In addition, chronic stress might evoke dendritic spine remodeling in perirhinal layer V pyramidal neurons.

## Materials and Methods

### Animals

To examine the protein expression of nectin-1 and nectin-3, 12-week-old male C57BL/6N mice were used (SLAC Laboratories, Shanghai, China). Male *Crhr1*^loxP/loxP^; *Camk2a-cre* mice with postnatal inactivation of the *Crhr1* gene in forebrain principal neurons (referred to as corticotropin-releasing hormone receptor 1 conditional knockout, CRHR1-CKO) and *R26*^flopCrh/flopCrh^; *Camk2a-cre* mice with postnatal overexpression of the *Crh* gene in forebrain principal neurons (referred to as corticotropin-releasing hormone conditional overexpression, CRH-COE) were generated as described previously (Müller et al., 2003; Lu et al., 2008). Both CRHR1-CKO and CRH-COE mice were kept on a mixed 129S2/Sv × C57BL/6J background. To visualize layer V pyramidal neurons in the perirhinal cortex, 8-week-old male Thy1-EYFP-H mice (stock number 003782; EYFP, enhanced yellow fluorescent protein) were purchased from the Jackson Laboratory (Bar Harbor, ME, USA). After arrival, mice were singly housed and habituated in the vivarium for 8 weeks before the start of experiment. Adult male CD1 mice were used as aggressors (Charles River Laboratories, Wilmington, MA, USA).

All animals were housed under a 12:12 h light/dark cycle (lights on at 07:00) and constant temperature (22 ± 1°C) with free access to food and water. The protocols were approved by the Animal Advisory Committee at Zhejiang University and the Committee for the Care and Use of Laboratory Animals of the Government of Upper Bavaria, Germany. All experiments were performed in accordance with the National Institute of Health’s Guide for the Use and Care of Laboratory Animals and European Communities Council Directive 2010/63/EU.

### Experimental Design

**Figure 1:**
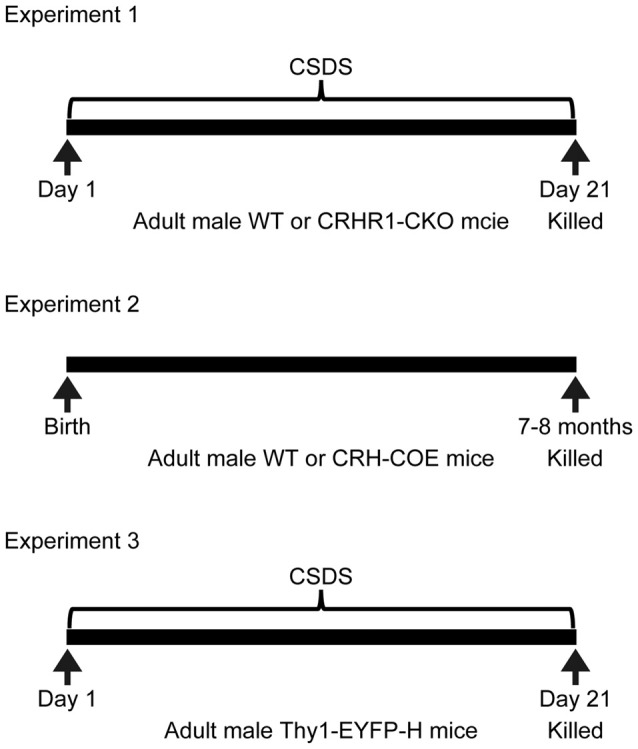
Experimental design. CRHR1-CKO, corticotropin-releasing hormone receptor 1-conditional knockout; CRH-COE, corticotropin-releasing hormone-conditional overexpression; CSDS, chronic social defeat stress; EYFP, enhanced yellow fluorescent protein; WT, wild-type.

The design of experiments was summarized in Figure 1. Experiment 1 examined the interactions between chronic stress and forebrain CRHR1 on the mRNA levels of nectin-1, nectin-3 and neurexin-1. Mouse brain samples from one previous study (Wang et al., 2011a) were used. These mice were 3–4.5 months old and tested in the novel object recognition task and the Y-maze task during the last week of chronic stress exposure. Mice were killed at 20 h after the last defeat episode (control wild-type (WT), *n* = 7; control CRHR1-CKO, *n* = 6; stressed WT, *n* = 8; stressed CRHR1-CKO, *n* = 7). Experiment 2 examined the effects of forebrain CRH overexpression on mRNA expression of nectin-1, nectin-3 and neurexin-1. Mouse brain samples from another previous study (Wang et al., 2011b) were used. These mice were 7–8 months old and tested in the Y-maze task and the Morris water maze task. Mice were killed under basal conditions (WT, *n* = 9; CRH-COE, *n* = 9). Experiment 3 examined the effects of chronic stress on dendritic spine plasticity in perirhinal layer V pyramidal neurons. Control and stressed Thy1-EYFP-H mice (*n* = 4 per group) were killed at 24 h after the last defeat episode. In addition, adult male C57BL/6N mice (*n* = 3) were used to examine the protein expression patterns of nectin-1 and nectin-3 in the perirhinal cortex under basal conditions.

### Chronic Social Defeat Stress Paradigm

The CSDS paradigm was applied as previously described (Wang et al., 2011a; Wagner et al., 2015). Adult male CD1 mice with a body weight over 40 g were housed in the defeat cages and their dominant behavior was trained with young C57BL/6N males. The defeat procedure was carried out between 12:00 and 16:00. Over 21 days, mice were daily introduced into the home cage of a different dominant CD1 mouse. All CD1 residents rapidly recognized and attacked the intruders within 2 min. To avoid serious injuries, the subordinate mouse was exposed to the CD1 aggressor for 30 s after being defeated. After the aggressive encounter, mice were separated by a holed metal partition, allowing the animals to keep continuous sensory but not physical contact for the next 24 h. Control mice were single-housed in their home cages and allowed to explore the empty defeat cages for 30 s daily.

### *In Situ* Hybridization

For Experiments 1 and 2, serial coronal sections were cut at 16 μm through the dorsal hippocampus (Bregma −1.58 to −2.18) in a cryotome (Microm HM 560, Thermo Fisher Scientific, Germany). The sections were thaw-mounted on superfrost slides and dried. *In situ* hybridization using ^35^S UTP-labeled ribonucleotide probes was performed as previously described (Schmidt et al., 2007). The following primers were used to generate an antisense cRNA hybridization probe (405 base pairs) that recognizes nectin-1: forward, GGCCATCTACAACCCGACTA; reverse, GTCATCAGCCGTTACCGTTT (Maurin et al., 2013). The riboprobe for nectin-3 variants (485 base pairs) was generated using the following primers: forward, AGCCGTTACATTCCCACTTG; reverse, ATTGT CCATCCAACCTGCTC (Wang et al., 2011a). The riboprobe for neurexin-1 (469 base pairs) was generated using the following primers: forward, AGTTGTACCTGGGTGGCTTG; reverse, TCACACGTCCTGCATCTAGC (Wang et al., 2011b). After radiolabeling, slides were apposed to Kodak Biomax MR films (Eastman Kodak, Rochester, NY, USA) and developed. Autoradiographs were digitized. Relative mRNA expression in the perirhinal cortex as well as the dentate gyrus (DG) and the retrosplenial cortex (RSC) was determined by optical densitometry (Scion Image, Scion, Frederick, MD, USA). For each mouse, 5–6 sections were analyzed.

### Brain Tissue Preparation and Immunostaining

To examine the protein expression profiles of nectin-1 and nectin-3 in the perirhinal cortex, adult male C57BL/6N mice were anesthetized by sodium pentobarbital (200 mg/kg of body weight, i.p.) and transcardially perfused with 0.9% saline followed by 4% paraformaldehyde (PFA) in 0.1 M phosphate-buffered saline (PBS, pH 7.4). Following post-fixation and cryoprotection, serial coronal sections (40 μm thick) were cut throughout the perirhinal cortex at −20°C in a cryostat (Leica, Wetzlar, Germany). The following primary antibodies were used for immunostaining: rabbit anti-nectin-1 (1:250, sc-28639, Santa Cruz Biotechnology, Santa Cruz, CA, USA), rabbit anti-nectin-3 (1:1000, ab63931, Abcam, Cambridge, UK), mouse anti-rat pyramidal cell (RPC; 1:5000, 345, Swant, Marly, Switzerland), mouse anti-glutamic acid decarboxylase 67 (GAD67; 1:500, MAB5406, Millipore, Bedford, MA, USA), and mouse anti-calbindin (1:2000, 300, Swant). The specificity of antibodies against nectin-1, nectin-3 and calbindin has been validated in previous studies (Fantin et al., 2013; Wang et al., 2013; Li et al., 2017). For immunohistochemistry, free-floating sections were treated with 3% hydrogen peroxide for 10 min followed by 1% normal goat serum for 1 h, and were then labeled with primary antibodies overnight at 4°C. The next day, sections were rinsed and incubated with a biotinylated goat anti-rabbit secondary antibody (Zhongshan Golden Bridge Biotechnology, Beijing, China) for 3 h at room temperature. After rinsing, the 3,3′-Diaminobenzidine Horseradish Peroxidase Color Development Kit (Zhongshan Golden Bridge) was used for staining. For double-labeling immunofluorescence, sections were treated with 1% normal donkey serum for 1 h and incubated with primary antibodies overnight at 4°C. The next day, sections were rinsed and labeled with the Alexa Fluor 488 donkey anti-rabbit and Alexa Fluor 594 donkey anti-mouse secondary antibodies (1:2000, Invitrogen, Carlsbad, CA, USA) for 3 h at room temperature. After rinsing, sections were transferred onto slides and coverslipped with Vectashield that contains 4′,6-diamidino-2-phenylindole (Vector Laboratories, Burlingame, CA, USA).

For Experiment 3, Thy1-EYFP-H mice were anesthetized by sodium pentobarbital and transcardially perfused by 1% buffered PFA followed by 4% PFA with 0.125% glutaraldehyde in 0.1 M PBS. Brains were post-fixed in the same fixative and cryoprotected. Horizontal sections (50 μm thick) were cut in a cryostat. After incubation with the goat anti-EYFP antibody (1:5000, ab5450, Abcam) overnight at 4°C, free-floating sections were rinsed and labeled with the Alexa Fluor 488 donkey anti-goat antibody (1:500, Invitrogen) for 3 h at room temperature. After rinsing, sections were transferred onto slides and coverslipped with Vectashield (Vector laboratories) to facilitate the delineation of the perirhinal cortex.

### Image Acquisition and Analysis

The regions of interest included perirhinal cortex areas 36 and 35 (Beaudin et al., 2013). To measure the immunoreactivity of nectin-1 and nectin-3, images from six sections per animal were acquired at 100× with a DP72 camera fitted to an Olympus BX61 microscope (Olympus, Tokyo, Japan) and analyzed with the NIH ImageJ software (National Institute of Health, Bethesda, MD, USA). Protein levels were determined by the differences in optical density values between the region of interest and the corpus callosum, which generally lacks staining and was considered as the background.

For perirhinal layer V pyramidal neurons, the initial dendritic segment is devoid of spines (DeFelipe and Fariñas, 1992) and the distal dendritic branches were usually truncated in 50-μm-thick sections. Therefore, the main apical dendrite (1 dendrite per neuron and 5–7 neurons per mouse) and oblique apical dendrites (1–2 dendrites per neuron and 5–7 neurons per mouse) with a distance of 100–200 μm from the center of the soma were digitized. The details about the total number and average length of selected dendrites were summarized in Supplementary Table S1. Images (1024 × 1024 pixels) were obtained using a Nikon Eclipse Ti microscope equipped with a Nikon A1R confocal system (Nikon, Tokyo, Japan). A 10× objective (NA 0.45), a 20× objective (NA 0.75) and a 60× oil-immersion objective (NA 1.40) were used. Dendrites were scanned at 0.2 μm intervals along the z-axis using the 60× objective with a 2.5× digital zoom, yielding a voxel size of 0.08 × 0.08 × 0.2 μm^3^. All dendrites were imaged at full dynamic range, and the pinhole size was set to 0.5 Airy Unit.

Image deconvolution was performed with the Huygens 4.3 software (Scientific Volume Imaging, Hilversum, Netherlands) as described previously (Heck et al., 2012). The settings were used so background intensity was averaged from the voxels with lowest intensity, and the signal-to-noise ratio was set to 12.5. After deconvolution, image brightness and contrast were adjusted using ImageJ. The density, volume, head diameter, and length of dendritic spines were analyzed with the NeuronStudio software^1^ (Dumitriu et al., 2011; Heck et al., 2012) by an investigator blind to the experimental conditions. For spine density analysis, data were processed by first averaging all dendritic segments from each mouse, followed by averaging all mice in each group. For spine morphology analysis, cumulative distributions included all automatically detected spines in a given group.

### Statistical Analysis

SPSS 16.0 (SPSS Inc., Chicago, IL, USA) and GraphPad Prism 5.0 (GraphPad Software Inc., San Diego, CA, USA) were used to perform statistical analysis. The normality of data distribution was assessed by the Shapiro-Wilk test and the homogeneity of variances was examined by Levene’s test. To compare the immunoreactivity of nectin-1 and nectin-3 in different layers of areas 36 and 35, one-way repeated measures analysis of variance (ANOVA) was performed, with the distance to the Bregma as the repeated within-subject factor and the brain region as the between-subject factor. To examine the effects of chronic stress on the mRNA levels of CAMs in WT and CRHR1-CKO mice, data were analyzed by two-way ANOVA (stress × genotype) followed by Bonferroni *post hoc* test when a significant interaction was observed. Student *t*-test was used to compare pairs of means. Mann-Whitney *U* test was used to compare the difference in the volume, head diameter and length of spines between groups. The level of statistical significance was set at *p* < 0.05. Data are expressed as mean ± standard error of the mean.

## Results

### Expression Profiles of Nectin-1, Nectin-3 and Neurexin-1 in the Perirhinal Cortex

**Figure 2:**
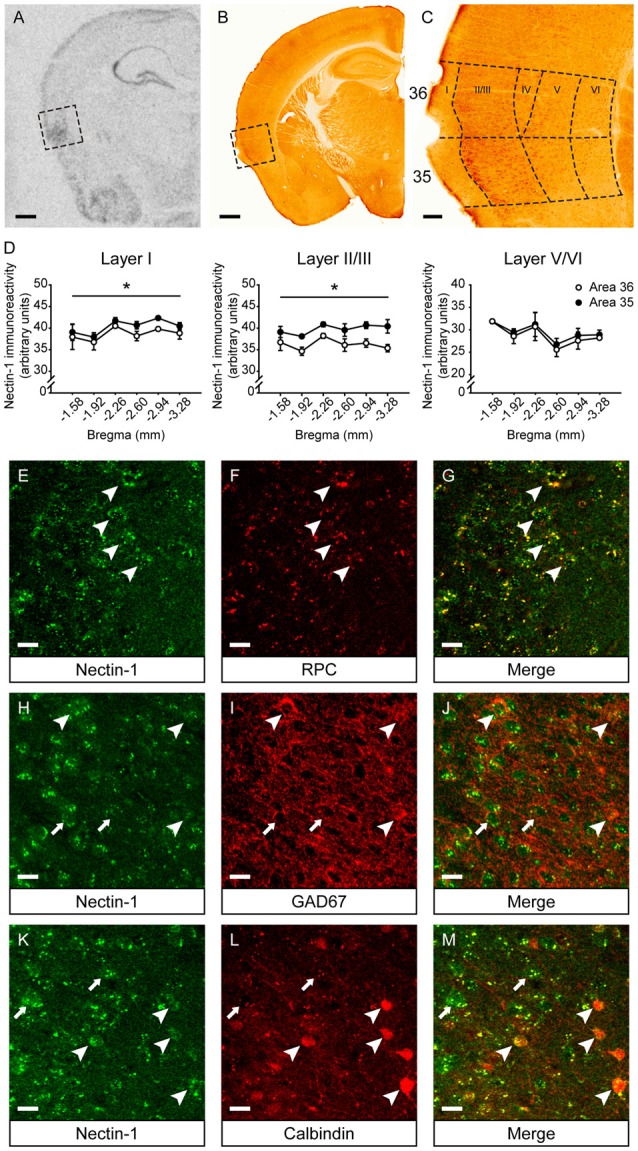
Nectin-1 mRNA and protein expression patterns in the adult mouse perirhinal cortex. **(A)** An *in situ* hybridization image showing nectin-1 mRNA expression in the perirhinal cortex (the boxed region) and adjacent brain regions. Scale bar = 500 μm. **(B)** A representative image showing nectin-1 immunostaining in the perirhinal cortex (the boxed region) and adjacent brain regions. Scale bar = 500 μm. **(C)** The magnified image of the insert in **(B)** showing nectin-1-positive cells in layers II–VI of areas 36 and 35. Scale bar = 50 μm. **(D)** Analysis of nectin-1 immunoreactivity in layers I–VI of perirhinal areas 36 and 35. *p < 0.05, main effect. *n* = 3 mice. **(E–G)** Nectin-1 colocalized with rat pyramidal cell (RPC), a marker for excitatory pyramidal neurons. Arrowheads indicate representative area 35 neurons co-immunostained with nectin-1 and RPC. **(H–J)** Nectin-1 partially colocalized with glutamic acid decarboxylase 67 (GAD67), a marker for inhibitory interneurons. Arrowheads indicate area 35 neurons that co-express nectin-1 and GAD67. Arrows indicate representative nectin-1-immunoreactive neurons surrounded by GAD67-positive inhibitory boutons. **(K–M)** Nectin-1 partially colocalized with calbindin, a marker for a subtype of pyramidal neurons and interneurons. Arrowheads indicate area 35 neurons co-immunostained with nectin-1 and calbindin. Arrows indicate representative nectin-1-positive neurons without calbindin expression. Scale bars for **(E–M)** are 20 μm.

First, we examined the mRNA and protein expression patterns of nectin-1 in the adult mouse perirhinal cortex. Nectin-1 mRNA was abundantly expressed in the perirhinal cortex (Figure 2A). Nectin-1-immunoreactive cells were observed in layers II–VI of the perirhinal cortex (Figures 2B,C and Supplementary Figures S1A–F). In layers I–III, nectin-1 immunoreactivity was higher in area 35 than in area 36 (layer I, *F*_(1,4)_ = 12.371, *p* = 0.025; layer II/III, *F*_(1,4)_ = 10.854, *p* = 0.030; one-way repeated measures ANOVA; Figure 2D). In addition, nectin-1 immunoreactivity in layers I–III was significantly higher than in layer V/VI (area 36—layer I vs. layer V/VI, *F*_(1,4)_ = 109.499, *p* = 0.00047; layer II/III vs. layer V/VI, *F*_(1,4)_ = 48.652, *p* = 0.002; area 35—layer I vs. layer V/VI, *F*_(1,4)_ = 223.252, *p* = 0.00012; layer II/III vs. layer V/VI, *F*_(1,4)_ = 89.516, *p* = 0.0007; one-way repeated measures ANOVA). No difference in perirhinal nectin-1 immunoreactivity at the rostrocaudal level was observed. We further found that perirhinal nectin-1 partially colocalized with RPC (a pyramidal neuron marker; Figures 2E–G and Supplementary Figures S1G–I), GAD67 (a general marker for interneurons; Figures 2H–J and Supplementary Figures S1J–L), and calbindin (a marker for a subtype of pyramidal neurons and interneurons; Figures 2K–M and Supplementary Figures S1M–O), indicating that nectin-1 is expressed by both excitatory pyramidal neurons and inhibitory interneurons.

Next, we examined nectin-3 mRNA and protein expression profiles in the perirhinal cortex (Supplementary Figures S2A–C). Nectin-3 mRNA and nectin-3-immunoreactive cells were present throughout perirhinal layers II–VI. In each layer, nectin-3 immunoreactivity between area 36 and area 35 was comparable (Supplementary Figure S2D). In area 36, nectin-3 immunoreactivity in layers I–III was higher than in layer V/VI (layer I vs. layer V/VI, *F*_(1,4)_ = 13.711, *p* = 0.021; layer II/III vs. layer V/VI, *F*_(1,4)_ = 7.869, *p* = 0.049; one-way repeated measures ANOVA). No significant difference in perirhinal nectin-3 immunoreactivity at the rostrocaudal level was found. In addition, neurexin-1 mRNA was enriched in layers II–VI of the perirhinal cortex (Supplementary Figure S2E). However, because of the lack of a specific antibody, neurexin-1 immunostaining was not performed.

### Chronic Stress Reduced Nectin-1 mRNA Levels in the Perirhinal Cortex Through the CRH-CRHR1 System

**Figure 3:**
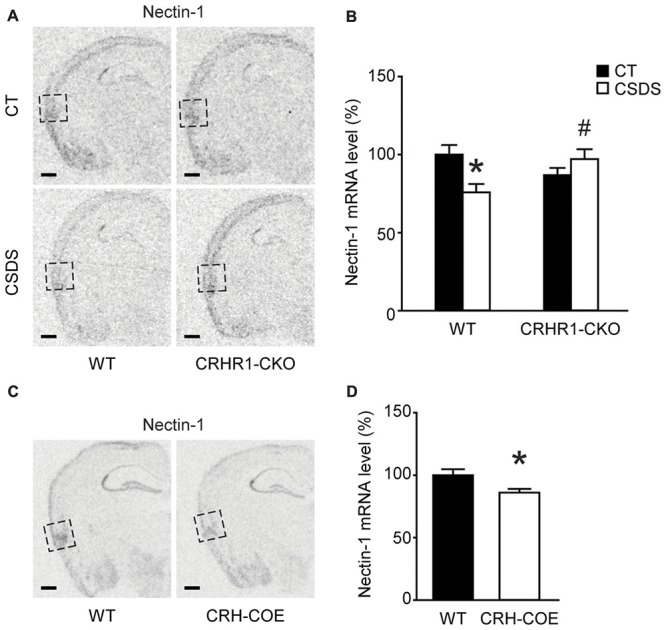
Effects of CSDS and the CRH-CRHR1 system on nectin-1 mRNA levels in the perirhinal cortex. **(A)** Representative *in situ* hybridization images showing nectin-1 mRNA expression in the perirhinal cortex (boxed regions) of WT or CRHR1-CKO mice with or without chronic stress exposure. **(B)** Nectin-1 mRNA levels in the perirhinal cortex were reduced by chronic stress in WT but not CRHR1-CKO mice. *n* = 6–8 mice per group. **(C)** Representative *in situ* hybridization images showing nectin-1 mRNA expression in the perirhinal cortex (boxed regions) of WT or CRH-COE mice. **(D)** Perirhinal nectin-1 mRNA levels were reduced in CRH-COE mice. *n* = 9 mice per group. All scale bars = 500 μm. *p < 0.05 vs. the control WT group; ^#^*p* < 0.05 vs. the stressed WT group.

We applied the CSDS paradigm and examined the effects of chronic stress on the mRNA levels of nectin-1, nectin-3 and neurexin-1 in the perirhinal cortex of WT and CRHR1-CKO mice. Moreover, the mRNA levels of these CAMs in the DG and the RSC, which are reciprocally connected with the perirhinal cortex and important for memory (Furtak et al., 2007; Agster and Burwell, 2009), were also measured. A significant stress × genotype interaction on nectin-1 mRNA levels in the perirhinal cortex was revealed (*F*_(1,24)_ = 9.396, *p* = 0.005, two-way ANOVA; Figures 3A,B). Perirhinal nectin-1 mRNA levels were markedly reduced by chronic stress in WT mice (control WT vs. stressed WT, *p* < 0.05, Bonferroni’s test), which were normalized by conditional knockout of forebrain CRHR1 (stressed CRHR1-CKO vs. stressed WT, *p* < 0.05, Bonferroni’s test). In addition, a significant genotype effect on nectin-1 mRNA levels was found in the RSC (*F*_(1,24)_ = 7.796, *p* = 0.0101, two-way ANOVA), but no significant main effects or a stress × genotype interaction were observed on nectin-1 levels in the DG (Table 1).

**Table 1:** Effects of chronic social defeat stress (CSDS) on the mRNA expression levels of nectin-1, nectin-3 and neurexin-1 in adult WT and corticotropin-releasing hormone receptor 1 conditional knockout (CRHR1-CKO) mice.

Brain region	mRNA	Group			
		CT-WT	CSDS-WT	CT-CKO	CSDS-CKO
PRH	Nectin-1	100 ± 6.19	75.89 ± 5.3*	86.91 ± 4.54	97.20 ± 6.23^#^
	Nectin-3	100 ± 20.08	119.10 ± 20.52	97.13 ± 7.64	99.83 ± 10.05
	Neurexin-1	100 ± 5.67	107.13 ± 7.45	96.72 ± 5.22	106.16 ± 8.97
DG	Nectin-1	100 ± 7.43	78.12 ± 11.98	93.47 ± 6.81	105.41 ± 10.10
	Nectin-3	100 ± 34.78	67.78 ± 11.96	83.53 ± 18.42	65.87 ± 6.82
	Neurexin-1	100 ± 2.52	103.61 ± 5.0	100.94 ± 5.62	105.15 ± 9.26
RSC	Nectin-1	100 ± 6.39	99.81 ± 13.54	113.49 ± 8.15	146.86 ± 11.70
	Nectin-3	100 ± 19.02	100.12 ± 16.8	112.70 ± 12.73	89.27 ± 10.07
	Neurexin-1	100 ± 5.45	117.81 ± 9.55	108.85 ± 8.08	124.24 ± 12.46

Gene expression levels in the perirhinal cortex (PRH), dentate gyrus (DG) and retrosplenial cortex (RSC) in each group are summarized. CT, control; WT, wild-type; CSDS, chronic social defeat stress; CKO, conditional knockout (of CRHR1). Data are expressed as mean ± standard error of the mean. *p < 0.05 vs. the control WT group; ^#^p < 0.05 vs. the stressed WT group. *n* = 6–8 mice per group.

To further assess the role of the CRH-CRHR1 system in the stress effects on perirhinal nectin-1 expression, CRH-COE mice with conditional forebrain CRH overexpression were used. Compared with the WT controls, CRH-COE mice had lower nectin-1 mRNA levels in the perirhinal cortex (*t*_(16)_ = 2.484, *p* < 0.05, unpaired *t*-test; Figures 3C,D). Nectin-1 mRNA levels were also reduced in the RSC (*t*_(16)_ = 2.618, *p* = 0.019, unpaired *t*-test) but not the DG of CRH-COE mice (Table 2). For nectin-3 and neurexin-1 mRNA levels in all brain regions examined, no significant difference among groups was found (Tables 1, 2). Taken together, these data suggest that chronic stress downregulates perirhinal nectin-1 mRNA levels via the CRH-CRHR1 system.

**Table 2:** The mRNA expression levels of nectin-1, nectin-3 and neurexin-1 in adult wild-type (WT) and corticotropin-releasing hormone conditional overexpression (CRH-COE) mice.

Brain region	mRNA	Group	
		WT	CRH-COE
PRH	Nectin-1	100 ± 4.77	86.03 ± 2.98*
	Nectin-3	100 ± 3.59	102.93 ± 3.49
	Neurexin-1	100 ± 3.80	105.40 ± 2.38
DG	Nectin-1	100 ± 3.61	98.30 ± 2.69
	Nectin-3	100 ± 10.5	87.53 ± 5.94
	Neurexin-1	100 ± 2.81	106.16 ± 2.59
RSC	Nectin-1	100 ± 7.60	72.08 ± 7.48*
	Nectin-3	100 ± 5.68	92.92 ± 3.78
	Neurexin-1	100 ± 3.17	102.79 ± 3.37

Gene expression levels in the perirhinal cortex (PRH), dentate gyrus (DG) and retrosplenial cortex (RSC) in wild-type (WT) and CRH-COE mice are summarized. Data are expressed as mean ± standard error of the mean. *p < 0.05 vs. the WT group. n = 9 mice per group.

### Chronic Stress Altered Spine Morphology in the Main Apical Dendrites and Reduced Spine Density in the Oblique Apical Dendrites of Perirhinal Layer V Pyramidal Neurons

**Figure 4:**
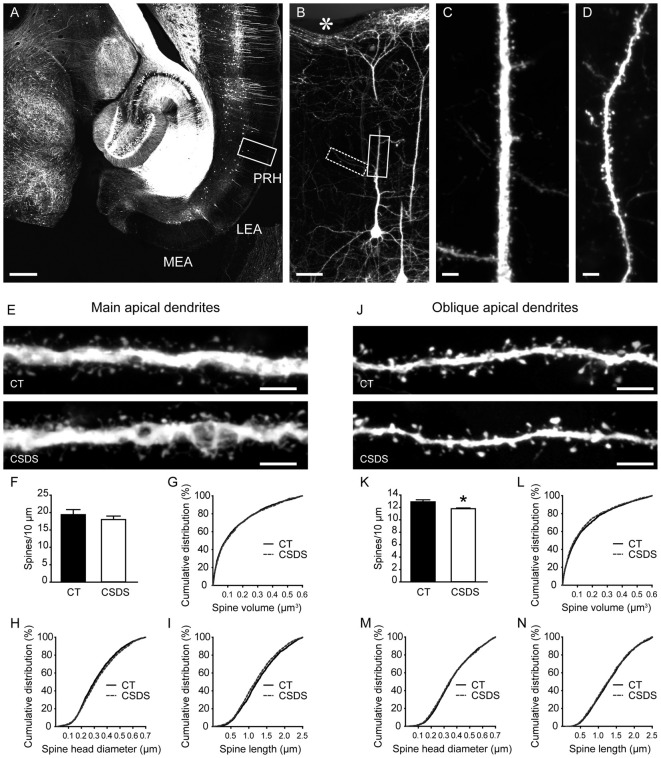
Effects of CSDS on dendritic spine plasticity in perirhinal layer V pyramidal neurons. **(A)** A transverse section at the ventral hippocampal level from a Thy1-EYFP-H mouse. Note that in the medial entorhinal cortex (MEA) and the lateral entorhinal cortex (LEA), very few neurons are labeled by EYFP. PRH, perirhinal cortex. Scale bar = 500 μm. **(B)** The magnified image of the insert in **(A)** showing EYFP-labeled layer V pyramidal neurons in the perirhinal cortex. The asterisk indicates the rhinal fissure. Scale bar = 50 μm. **(C)** High-resolution projected raw z-stacks of the main apical dendrite in the insert (solid line) in **(B)**. Scale bar = 5 μm. **(D)** High-resolution projected raw z-stacks of the oblique apical dendrite in the inset (dashed line) in **(B)**. Scale bar = 5 μm. **(E)** Representative deconvolved and projected z-stacks showing the main apical dendrites in the control and defeat mice. Scale bars = 5 μm. **(F,G)** In the main apical dendrites, spine density and spine volume were comparable between groups. **(H,I)** CSDS increased spine head diameter but reduced spine length. **(J)** Representative deconvolved and projected z-stacks showing the oblique apical dendrites in the control and defeat mice. Scale bars = 5 μm. **(K)** CSDS reduced spine density in the oblique apical dendrites of perirhinal layer V pyramidal neurons. **(L–N)** Spine volume, spine head diameter and spine length were similar between groups. *p < 0.05 vs. the control group. *n* = 4 mice per group.

To evaluate the influences of CSDS on dendritic spine plasticity in the perirhinal cortex, we used Thy1-EYFP-H mice with clear labeling of perirhinal layer V pyramidal neurons (Figures 4A–D). We first analyzed spines in the main apical dendrites of perirhinal layer V pyramidal neurons (Figure 4E). In chronically stressed mice, both the density and the volume of spines in the main apical dendrites were similar to the controls (density: *t*_(6)_ = 0.8078, *p* = 0.4501, unpaired *t*-test, Figure 4F; volume: 0.1483 ± 0.0033 vs. 0.1515 ± 0.0035, *p* = 0.36, Mann-Whitney *U* test, Figure 4G). Chronic stress slightly increased spine head diameter (0.3241 ± 0.0029 vs. 0.3343 ± 0.0032, *p* = 0.0205, Mann-Whitney *U* test; Figure 4H), but significantly reduced spine length (1.2886 ± 0.0113 vs. 1.2326 ± 0.0115, *p* = 0.0011, Mann-Whitney *U* test; Figure 4I), which may explain the overall unchanged spine volume in stressed mice.

We then analyzed the density and morphology of spines in the oblique apical dendrites of perirhinal layer V pyramidal neurons (Figure 4J). CSDS induced a subtle but significant reduction of spine density in the oblique apical dendrites (*t*_(6)_ = 3.139, *p* = 0.0201, unpaired *t*-test; Figure 4K). Spine volume, spine head diameter and spine length were comparable between control and stressed mice (volume: 0.1496 ± 0.0037 vs. 0.1415 ± 0.0036, *p* = 0.171; head diameter: 0.3685 ± 0.0034 vs. 0.3656 ± 0.0036, *p* = 0.4174; length: 1.315 ± 0.0121 vs. 1.2934 ± 0.0121, *p* = 0.224; Mann-Whitney *U* test; Figures 4L–N). Therefore, chronic stress impaired dendritic spine plasticity in perirhinal layer V pyramidal neurons.

## Discussion

The impact of chronic stress on hippocampal synaptic plasticity and hippocampus-dependent memory has received broad attention. In this study, we provided evidence that chronic stress reduced nectin-1 mRNA levels and induced dendritic spine remodeling in the perirhinal cortex, a parahippocampal structure interconnected with the hippocampus. In addition, the downregulation of perirhinal nectin-1 levels by chronic stress was modulated by the CRH-CRHR1 system. These chronic stress-induced molecular and cellular alterations in the perirhinal cortex may contribute to cognitive deficits.

Perirhinal neurons are activated when animals navigate through an environment with objects (Burke et al., 2012), and they play an essential role in various forms of recognition memory (Eichenbaum et al., 2007; Squire et al., 2007; Suzuki and Naya, 2014). In adult animals, chronic stress impairs performance in the novel object recognition task (Wang et al., 2011a; Barsegyan et al., 2015; Franklin et al., 2018) that is dependent on the perirhinal cortex (Dere et al., 2007). In addition, chronic stress impairs temporal order memory (van der Kooij et al., 2014) that requires the functional connections among the perirhinal cortex, the hippocampus and the prefrontal cortex (Barker et al., 2007; Barker and Warburton, 2011). In line with these behavioral observations, we found that chronic stress reduced the mRNA levels of nectin-1, a presynaptic CAM involved in synaptic formation and plasticity (Honda et al., 2006; Togashi et al., 2006; Fantin et al., 2013), and impaired dendritic spine plasticity in the perirhinal cortex. These behavioral and molecular data suggest that, similar to the hippocampus, the perirhinal cortex is also sensitive to the adverse effects of chronic stress.

Synaptic CAMs may modulate the effects of chronic stress on synaptic plasticity and cognition (Sandi, 2004). Among these molecules, nectin-3 and neurexin-1 are implicated in chronic stress-induced synaptic and cognitive impairments (Wang et al., 2011a,b, 2013; van der Kooij et al., 2014). We observed that nectin-1, nectin-3 and neurexin-1 were expressed in the perirhinal cortex. Notably, nectin-1, which forms heterophilic adhesion with postsynaptic nectin-3, was abundantly expressed in perirhinal neurons. Most importantly, nectin-1 mRNA levels were selectively reduced in the perirhinal cortex, but not the DG or the RSC, of chronically stressed WT mice. In comparison, the mRNA levels of nectin-3 and neurexin-1 remained unchanged in all brain regions examined in stressed WT mice. Considering the involvement of nectin-1 in synaptic plasticity, it is possible that such a reduction leads to structural and functional alterations in stressed mice. Collectively, these results highlight nectin-1 as a molecular target of chronic stress, and indicate that the regulation of CAM levels by chronic stress may be brain region-specific.

The CRH-CRHR1 system has been proposed to mediate the adverse effects of chronic stress on hippocampal structure and function (Chen et al., 2012; Maras and Baram, 2012). Chronic stress enhances hippocampal CRH-CRHR1 signaling, which in turn evokes dendritic retraction, spine elimination, reduced nectin-3 expression, and memory deficits (Wang et al., 2011a, 2013). We found that chronic stress suppressed perirhinal nectin-1 gene expression in WT but not CRHR1-CKO mice. Similar to stressed WT mice, CRH-COE mice had decreased nectin-1 mRNA levels in the perirhinal cortex. Our results indicate that chronic stress reduces nectin-1 levels through the CRH-CRHR1 system. Since both CRH and CRHR1 are expressed in the perirhinal cortex (Wong et al., 1994; Kühne et al., 2012), it is likely that chronic stress activates local CRH-CRHR1 system to exert such effects. Consistently, stressed WT mice with reduced perirhinal nectin-1 levels showed impaired novel object recognition memory and spatial memory, whereas stressed CRHR1-CKO mice with unchanged perirhinal nectin-1 levels had intact memory as we reported previously (Wang et al., 2011a). In comparison, CRH-COE mice with reduced perirhinal nectin-1 mRNA levels also showed impaired spatial learning and memory (Wang et al., 2011b). These data further strengthen the link between reduced perirhinal nectin-1 levels and impaired recognition memory.

The Thy1-EYFP-H mouse line shows clear labeling of neocortical layer V pyramidal neurons, whose cellular, molecular and electrophysiological properties have been characterized (Sugino et al., 2006; Miller et al., 2008; Yu et al., 2008; Porrero et al., 2010). We evaluated the effects of chronic stress on the density and morphology of dendritic spines in perirhinal layer V pyramidal neurons in these mice. In the main apical dendrites, spine dimensions were changed by chronic stress, resulting in increased spine head diameter but shortened spine length. This raises the possibility that chronic stress may increase the size of postsynaptic density surface in these neuron populations, similar to the effects of chronic stress on dendritic spines in the stratum lacunosum-moleculare of CA1 (Donohue et al., 2006). In the oblique apical dendrites, spine morphology remained unchanged after chronic stress, while a reduction in spine density was found in stressed mice. These results suggest a dendritic domain-specific modulation of spine plasticity. Although the dendritic domain-specific innervation of perirhinal layer V pyramidal neurons remains to be studied, these neurons receive inputs from various cortical regions and the hippocampus (Kealy and Commins, 2011). Viewing from a circuit perspective, dendritic spines implement a distributed circuit with widespread connectivity and endow the circuit with input-specific learning rules (Yuste, 2011). Therefore, impaired spine morphology and/or reduced spine density in perirhinal pyramidal neurons may disrupt information processing in the hippocampal-parahippocampal network and lead to cognitive deficits. The causal relationship among dysregulated nectin-1 levels, altered dendritic spine plasticity and impaired recognition memory needs to be investigated in future studies.

The current study has a few limitations. First, due to the availability of brain samples, we did not analyze the protein levels of CAMs and dendritic spine morphology in the perirhinal cortex of CRHR1-CKO and CRH-COE mice. Further studies are necessary to determine whether chronic stress reduces perirhinal CAM protein levels and impairs dendritic spine plasticity in a CRHR1-dependent manner. Second, CAM mRNA levels were examined in young adult CRHR1-CKO mice but in middle-aged CRH-COE mice. The potential interactions among age, stress and the CRH-CRHR1 system on CAM levels merit further investigations.

In summary, our study demonstrates that the perirhinal cortex is vulnerable to chronic stress. The observed molecular and structural abnormalities in the perirhinal cortex may underlie chronic stress-induced memory loss.

## Author Contributions

QG, Y-AS, CW and X-DW performed the experiments. QG and X-DW analyzed the data. JMD contributed analytic tools. T-MS, JMD, MVS and XDW drafted the manuscript.

## Conflict of Interest Statement

The authors declare that the research was conducted in the absence of any commercial or financial relationships that could be construed as a potential conflict of interest.

## Footnotes

^1^http://research.mssm.edu/cnic/tools-ns.html
