# Supplementary material for: Chronic Stress Reduces Nectin-1 mRNA Levels and Disrupts Dendritic Spine Plasticity in the Adult Mouse Perirhinal Cortex
Source: Front Cell Neurosci. 2018 Mar 13;12:67. doi: 10.3389/fncel.2018.00067 (PMC5859075; doi:10.3389/fncel.2018.00067)
Supplement: Supplementary file 1 [file Data_Sheet_1.DOC]

**Supplementary Information for:**

**Chronic Stress Reduces Nectin-1 mRNA Levels and Disrupts Dendritic Spine Plasticity in the Adult Mouse Perirhinal Cortex**

Qian Gong, Yun-Ai Su, Chen Wu, Tian-Mei Si, Jan M. Deussing, Mathias V. Schmidt, and Xiao-Dong Wang

**Supplementary Figures**

**
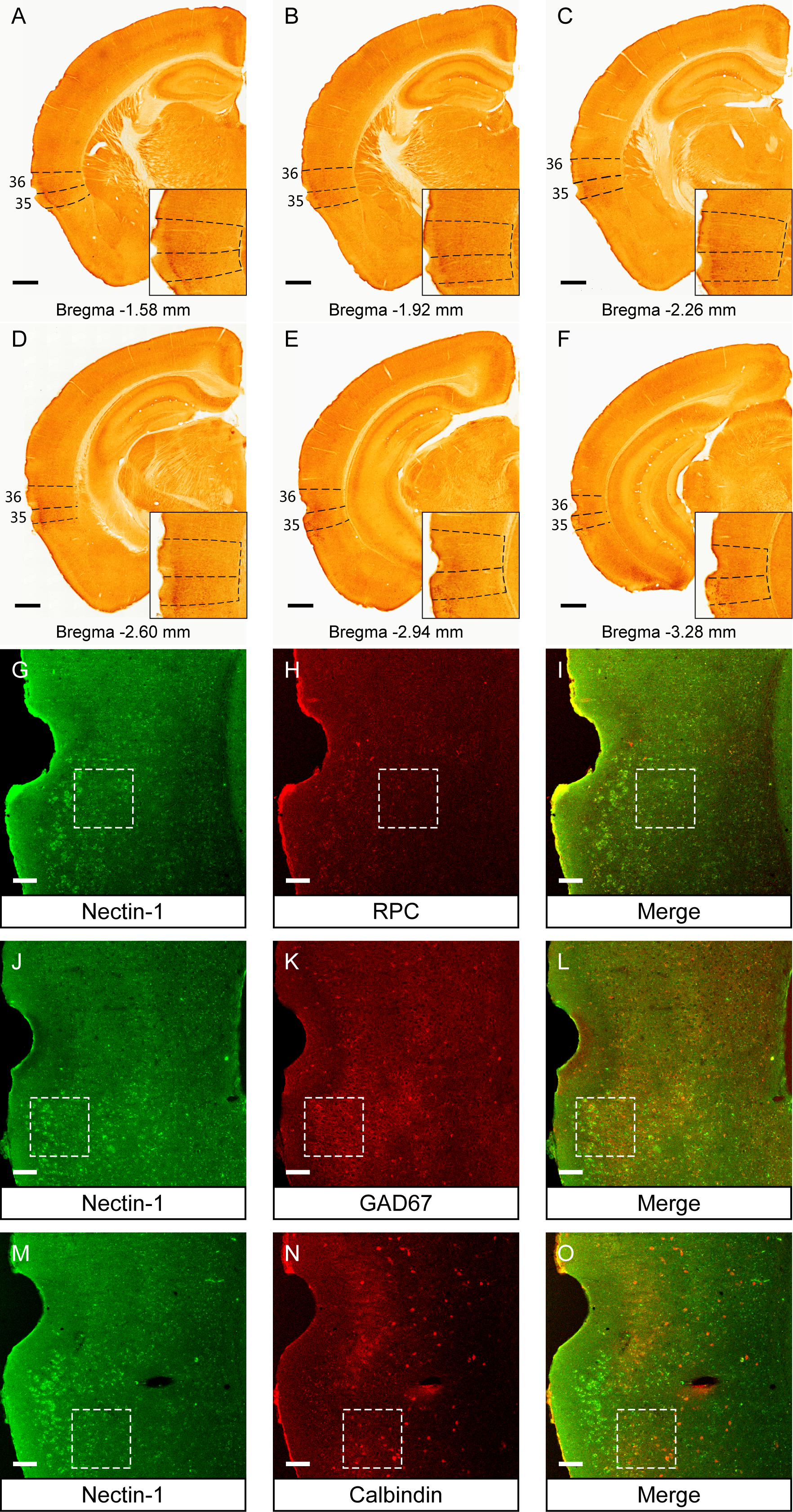
**

**Figure S1.** Nectin-1 protein expression patterns in the adult mouse perirhinal cortex. (A-F) Representative images showing nectin-1 immunostaining in perirhinal areas 36 and 35 at the rostrocaudal level. Inserts are magnified images showing nectin-1-immunoreactive cells in layers II-VI of areas 36 and 35. Scale bars = 500 µm. (G-I) Colocalization between nectin-1 and rat pyramidal cell (RPC) in areas 36 and 35. The boxed regions are magnified and presented in Figure 2E-2G. (J-L) Partial colocalization between nectin-1 and glutamic acid decarboxylase 67 (GAD67) in areas 36 and 35. The boxed regions are magnified and presented in Figure 2H-2J. (M-O) Partial colocalization between nectin-1 and calbindin in areas 36 and 35. The boxed regions are magnified and presented in Figure 2K-2M. Scale bars for G-O are 50 µm.

**
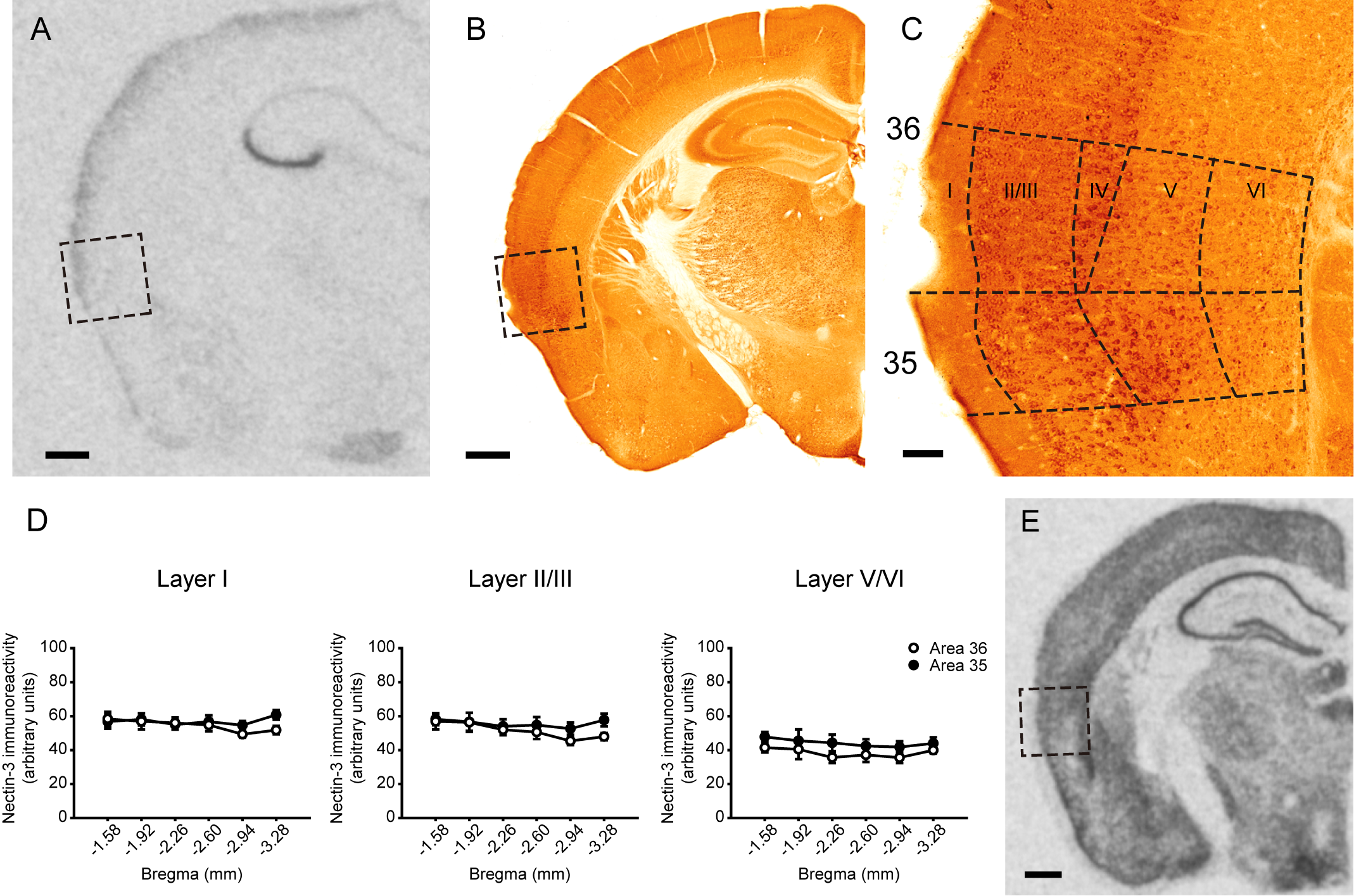
**

**Figure S2.** Nectin-3 and neurexin-1 expression patterns in the adult mouse perirhinal cortex. (A) An *in situ* hybridization image showing nectin-3 mRNA expression in the perirhinal cortex (the boxed region) and adjacent brain regions. Scale bar = 500 µm. (B) A representative image showing nectin-3 immunostaining in the perirhinal cortex (the boxed region) and adjacent brain regions. Scale bar = 500 µm. (C) The magnified image of the insert in (B) showing nectin-3-positive cells in layers II-VI of areas 36 and 35. Scale bar = 50 µm. (D) Analysis of nectin-3 immunoreactivity in layers I-VI of perirhinal areas 36 and 35. (E) An *in situ* hybridization image showing neurexin-1 mRNA expression in the perirhinal cortex (the boxed region) and adjacent brain regions. Scale bar = 500 µm.

**Table S1.** The total number and average length of dendritic segments selected for morphological analysis from perirhinal layer V pyramidal neurons in control or chronically stressed mice.

| **Dendritic domain** | **Number of dendrites** | | **Length of segments (µm)** | | |
| --- | --- | --- | --- | --- | --- |
|  | **CT** | **CSDS** | **CT** | **CSDS** | **Statistics** |
| Main apical dendrite | 28 | 31 | 628.44 ± 26.53 | 657.76 ± 10.76 | *t*_6_ = 1.024,  *p* = 0.345 |
| Oblique apical dendrite | 35 | 35 | 594.62 ± 73.28 | 638.16 ± 54.72 | *t*_6_ = 0.476,  *p* = 0.651 |

CT, control; CSDS, chronic social defeat stress. Data are expressed as mean ± standard error of the mean. n = 4 mice per group.
